# Supplementary material for: ZMYND8 mediated liquid condensates spatiotemporally decommission the latent super-enhancers during macrophage polarization
Source: Nat Commun. 2021 Nov 11;12:6535. doi: 10.1038/s41467-021-26864-x (PMC8586003; doi:10.1038/s41467-021-26864-x)
Supplement: Supplementary file 1 — Supplementary Information [file 41467_2021_26864_MOESM1_ESM.pdf]

Supplementary Fig. 1 *Zmynd8* deficiency does not affect the development and maturation of myeloid cells.

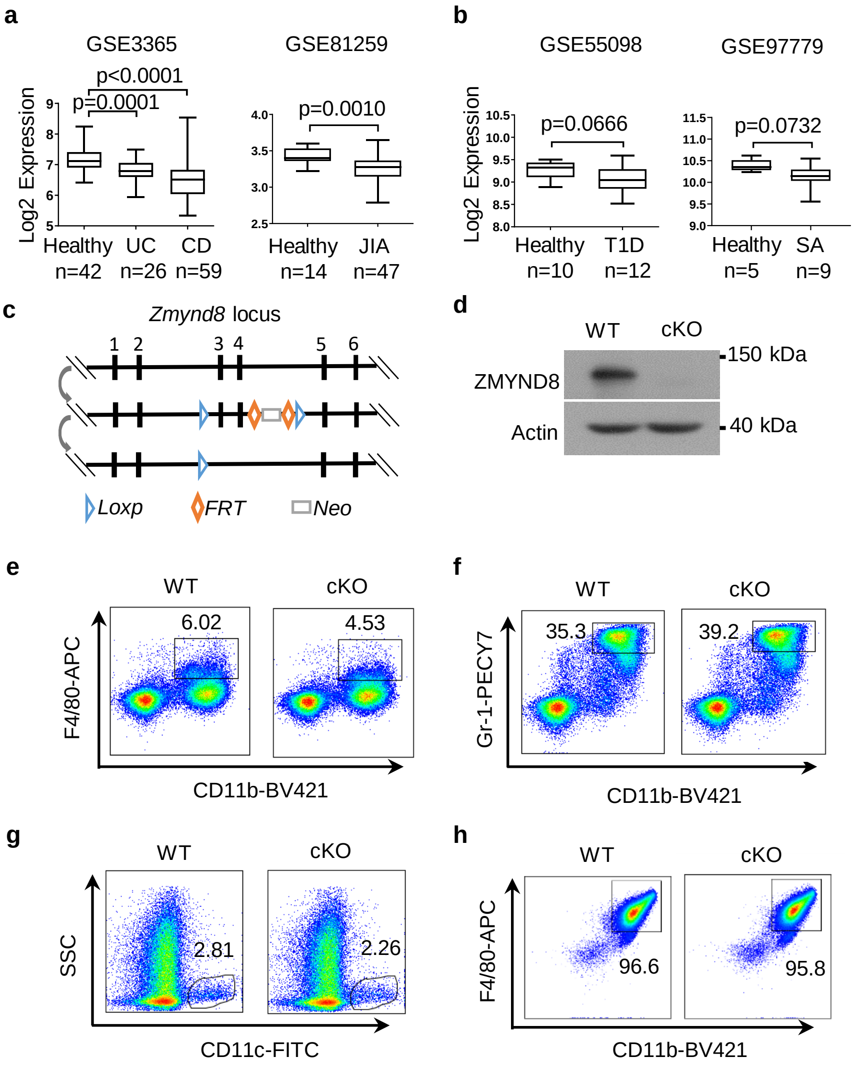

Supplementary Fig. 1 *Zmynd8* deficiency does not affect the development and maturation of myeloid cells.

(a-b) Analysis of *ZMYND8* expression in healthy and autoimmune diseases patients. UC: Ulcerative Colitis, CD: Crohn's disease, JIA: Juvenile Idiopathic Arthritis, T1D: Type 1 Diabetes, SA: Sebaceous Adenitis [46-49]. Whiskers: Min to Max, with a centerline indicating the median. Two-tailed Student's t test determined *p* values. **(c)** Scheme of targeting strategy of *Zmynd8* conditional knockout mice. **(d)** Immunoblot analysis of *Zmynd8* in BMDMs from *Zmynd8* $^{f/f}$ (WT) and *Zmynd8* $^{f/f}$; *LysM*-cre^{+} (*Zmynd8* cKO) mice. Experiments were repeated three times. **(e-g)** Flow cytometry analysis of CD11b^{+} F4/80^{+} macrophages (Ma) **(e)**, CD11b^{+} Gr-1^{+} neutrophils (Neu) **(f)**,and CD11c^{+} dendritic cells (DCs) **(g)** from bone marrow. **(h)** Flow cytometry of the surface expression of CD11b and F4/80 on *in vitro* differentiated BMDMs. Source data are provided as a Source Data file.

Supplementary Fig. 2 Zmynd8 repress pro-inflammatory genes expression in macrophage

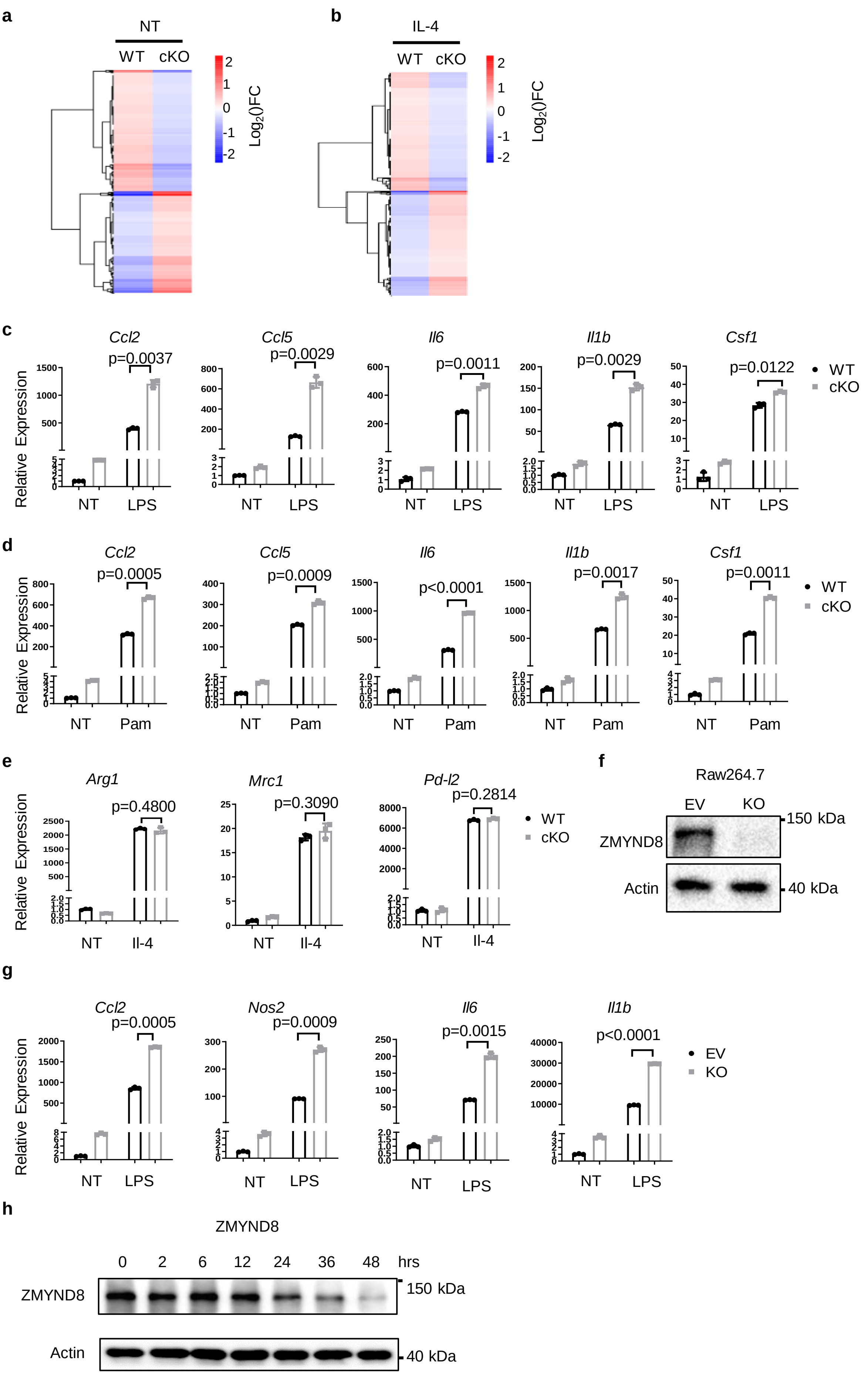

Supplementary Fig. 2 *Zmynd8* represses pro-inflammatory gene expression in macrophages.

(a-b) RNA-Seq analysis by using WT and *Zmynd8* cKO macrophages left nontreated (NT) **(a)** or stimulated with IL-4 for 6h **(b)**. Heatmap analysis showing differentially expressed genes with adjusted *p* value <0.05, false discovery rate <0.05, and log2 fold-change >1.2. **(c)** Relative expression of indicated proinflammatory genes in WT and *Zmynd8* cKO BMDMs treated with LPS for 6h was analyzed by qRT-PCR (n=3 biologically independent samples). All data are means ± SD. Two-tailed Student's t test determined *p* values. **(d)** qRT-PCR evaluates the relative expression of indicated proinflammatory genes in WT and *Zmynd8* cKO BMDMs treated with Pam3CSK4 for 6h (n=3 biologically independent samples). All data are means ± SD. Two-tailed Student's t test determined *p* values. **(e)** The relative expression of indicated genes in WT and *Zmynd8* cKO BMDMs treated with IL-4 for 6h was analyzed by qRT-PCR (n=3 biologically independent samples). All data are means ± SD. Two-tailed Student's t test determined *p* values. **(f)** The protein level of ZMYND8 in the Control (EV) and *Zmynd8* KO Raw264.7 cells were examined by western blot. **(g)** qRT-PCR measures the relative expression of indicated proinflammatory genes in Control (EV) and *Zmynd8* KO Raw264.7 cells treated with LPS for 6h (n=3 biologically independent samples). All data are means ± SD. Two-tailed Student's t test determined *p* values. **(h)** Immunoblotting of ZMYND8 protein level in WT BMDMs after LPS treatment. Source data are provided as a Source Data file.

Supplementary Fig. 3 *Zmynd8* does not influence the TLR4 signal transduction in macrophages

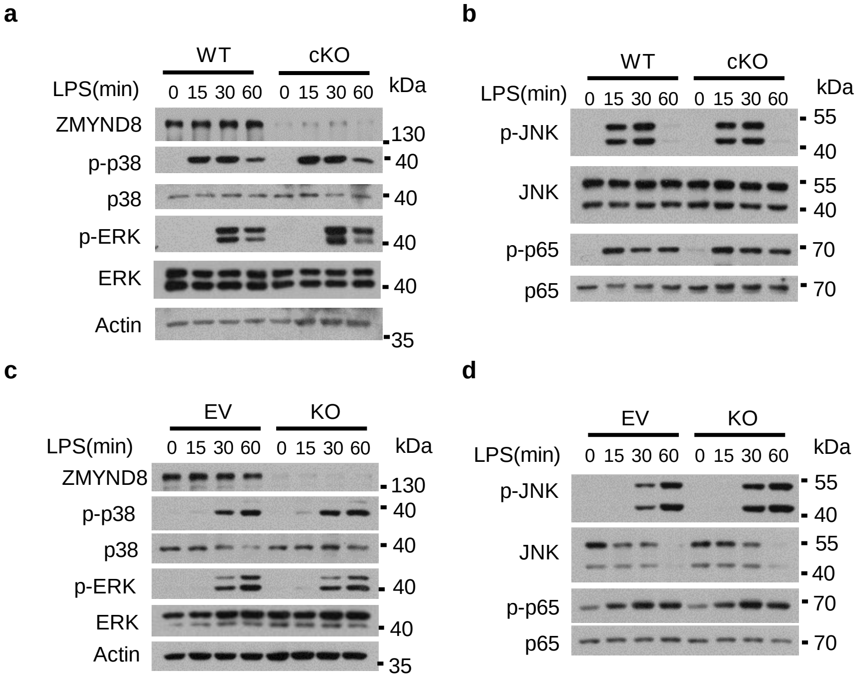

Supplementary Fig. 3 *Zmynd8* does not influence the TLR4 signal transduction in macrophages.

(a-b) Immunoblot analysis of phosphorylated (p-) and total p38, NF-κB/p65, and MAPK signaling proteins in whole-cell lysates of WT and *Zmynd8* cKO BMDMs left unstimulated and stimulated with LPS (100 ng/ml) at the indicated time points. **(c-d)** Immunoblot analysis of phosphorylated (p-) and total p38, NF-κB/p65, and MAPK signaling proteins in whole-cell lysates of control (EV) and *Zmynd8*-deficient (KO) Raw264.7 cells, which were stimulated with LPS (100 ng/ml) at the indicated time points. The experiments above were repeated three times. Source data are provided as a Source Data file.

Supplementary Fig. 4 ZMYND8 binding preference at the enhancer region.

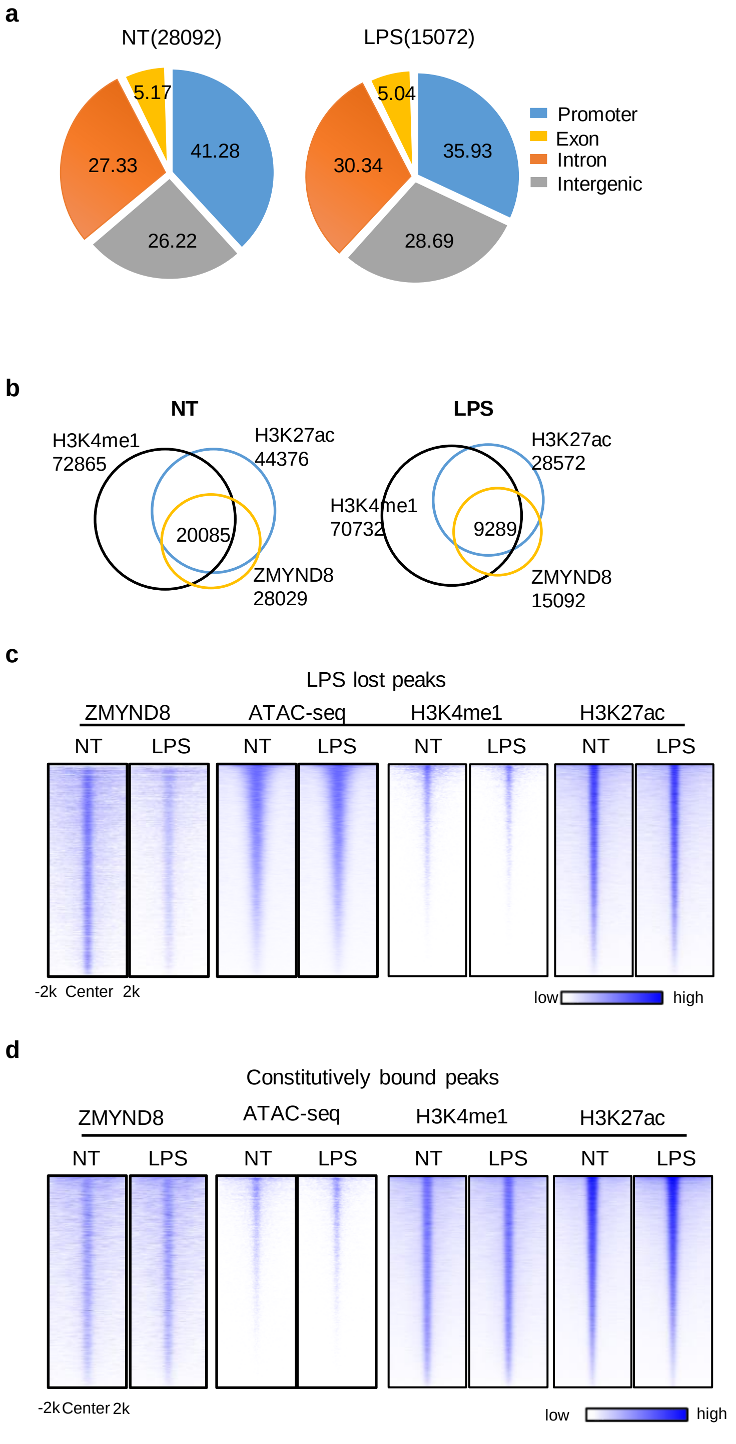

Supplementary Fig. 4 ZMYND8 binding preference at the enhancer region.

(a) The genomic distribution of ZMYND8 ChIP-seq peaks in NT (left) and LPS-treated (right) WT BMDMs. **(b)** Most ZMYND8 occupies the active enhancer regions marked with H3K27ac and H3K4me1 ChIP-seq data in NT (left) and classically polarized (right) BMDMs. **(c)** Heatmap analysis of ChIP-seq data obtained from NT and classically polarized BMDMs. Peak density heatmap showing H3K4me1, H3K27ac, and chromatin accessibility (ATAC-seq) signal at the regions where LPS-lost ZMYND8 peaks are located. **(d)** H3K4me1, H3K27ac, and chromatin accessibility (ATAC-seq) signal at the ZMYND8 constitutively bound region were also shown. Each row shows ±2kb centered regions of ZMYND8 peaks. Source data are provided as a Source Data file.

Supplementary Fig. 5 ZMYND8 IDR region is responsible for the formation of phase-separated liquid condensates in the nucleus.

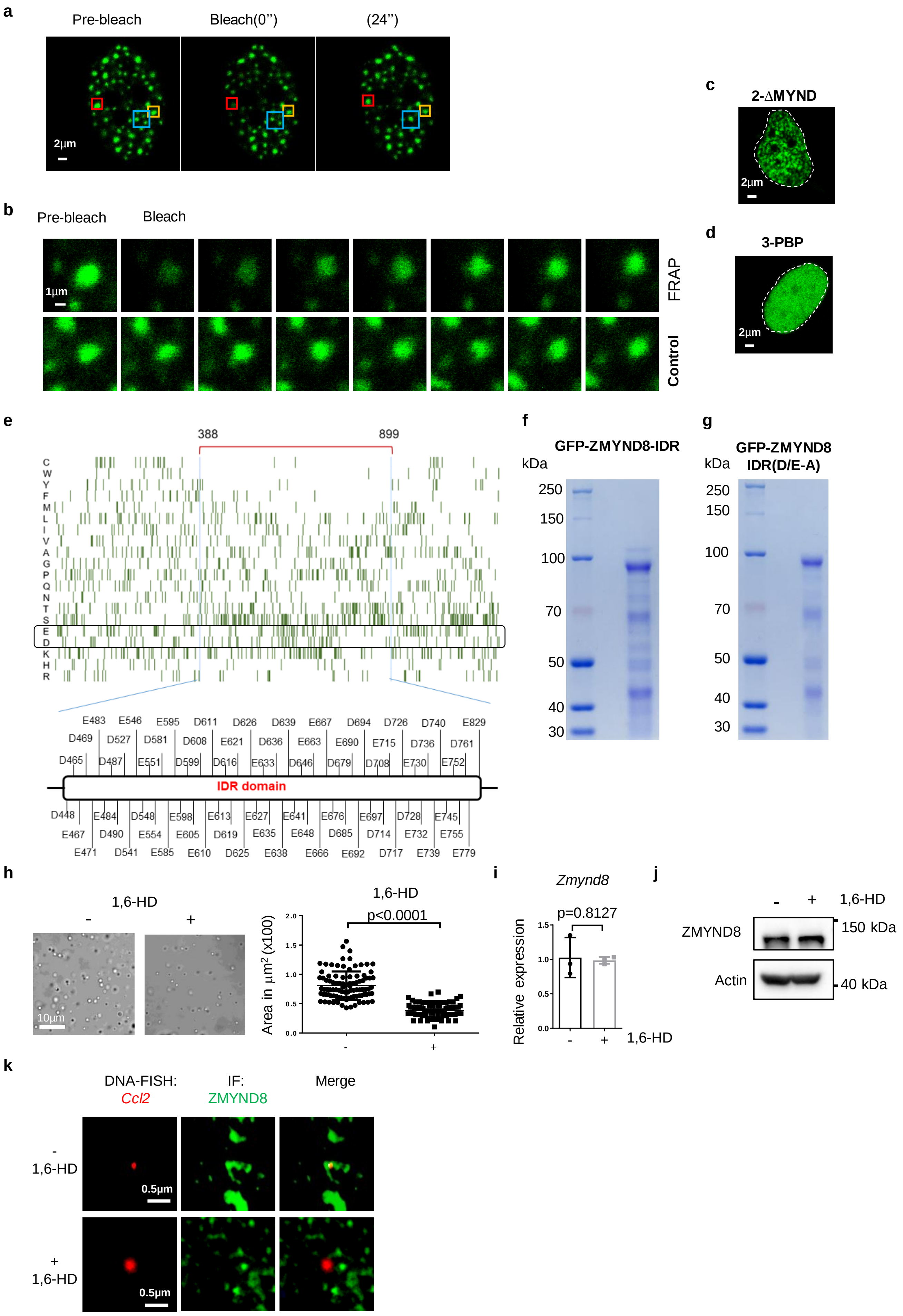

Supplementary Fig. 5 ZMYND8 IDR region is required for the phase-separated liquid condensates formation in the nucleus.

(a) Image of an intracellular GFP-ZMYND8-IDR optoDroplet (red box) before (left), during (middle) and after (right) photobleaching. The yellow box highlights an unbleached region for comparison. The time relative to photobleaching (0”) is indicated. The blue box highlighted the droplet fusion event, which was also shown in Fig. 3g. **(b)** Time-lapse and close-up view of droplet recovery for regions highlighted in **(a)**. The time relative to photobleaching is indicated. Scale bar, 1 μm. **(c-d)** Representative images of ZMYND8 constructs, 2-ΔMYND **(c)** and 3-PBP **(d)** overexpressed in HeLa cells. The experiments above were repeated three times. **(e)** Amino acid composition of the ZMYND8 protein. Each row represents information for a single amino acid, single-letter amino code shown on the left. The length of the row corresponds to the length of the ZMYND8 protein. The red bar represents the IDR of ZMYND8 under investigation. A total 66 Aspartic acids (D) and Glutamic acids (E) are present in the IDR region. **(f-g)** The C-terminal tagged GFP–ZMYND8-IDR fusion **(f)** and GFP-ZMYND8-IDR(D/E-A) **(g)** were expressed and purified from recombinant E. coli Rosetta (DE3) cells. Each fusion protein was examined by SDS–PAGE followed by Coomassie blue staining. Experiments were repeated three times. **(h)** Liquid droplets formed by full length ZMYND8 was treated with (+) (n=88 samples, examined over three independent experiments) or without (-) (n=94 samples, examined over three independent experiments) 1,6-Hexanediol (1,6-HD). Representative images (left) and statistic analysis of droplet size (right) were shown. Data are presented as mean values +/- SD. Two-tailed Student’s t-test determined *p* values. The mRNA (n=3 biologically independent samples) **(i)** and protein level **(j)** of ZMYND8 were measured in the presence (+) or absence (-) of 1,6-HD treatment. Data are presented as mean values +/- SD. Two-tailed Student’s t-test determined *p* values. **(k)** Co-localization between ZMYND8 protein and the *Ccl2* probe in fixed WT BMDMs treated with (+) or without (-) 1,6-HD was evaluated with IF followed by DNA-FISH. Experiments were repeated three times. Source data are provided as a Source Data file.

Supplementary Fig. 6 ZMYND8 liquid condensates coalesce with p65.

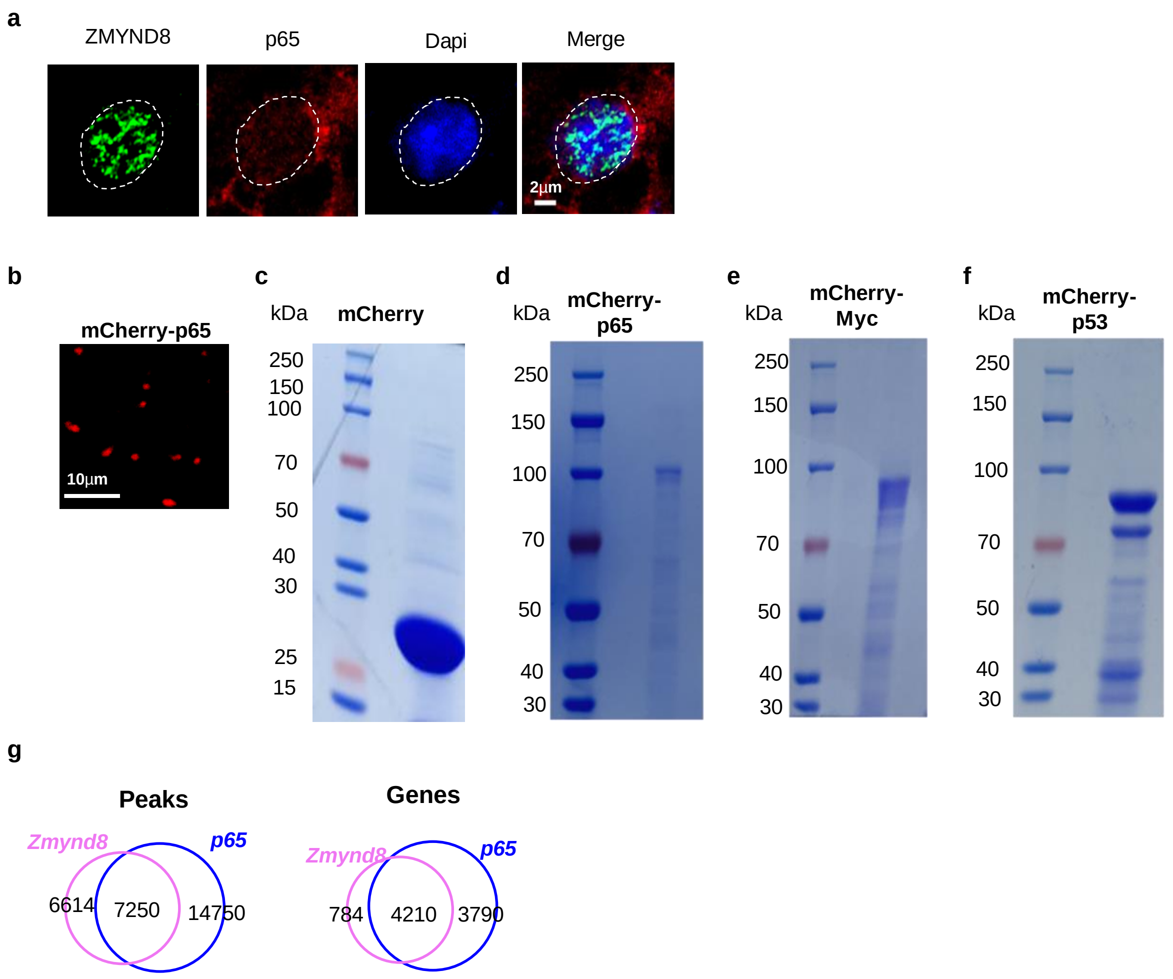

Supplementary Fig. 6 ZMYND8 liquid condensates coalesce with p65.

(a) Immunofluorescence staining of endogenous ZMYND8 (Green) in primary mouse BMDMs with endogenous p65 (Red) before treatment with LPS, DAPI nuclear staining in blue.**(b)** The representative image of mCherry-p65 liquid droplets. 10μM mCherry-p65 were added to droplet formation buffers with 125 mM NaCl and 10% PEG8000. **(c-f)** The mCherry **(c),** mCherry-p65 **(d),** mCherry-MYC **(e),** and mCherry-p53 **(f)** were expressed and purified from E. coli Rosetta (DE3) cells. Each fusion protein was examined by SDS–PAGE followed by Coomassie blue staining. The experiments above were repeated three times. **(g)** Venn diagram of the overlapped peaks (left) and genes (genes) between ZMYND8 and p65 in classically polarized BMDMs. Source data are provided as a Source Data file.

Supplementary Fig. 7 K122 acetylated p65 facilitates the dynamic redistribution of ZMYND8.

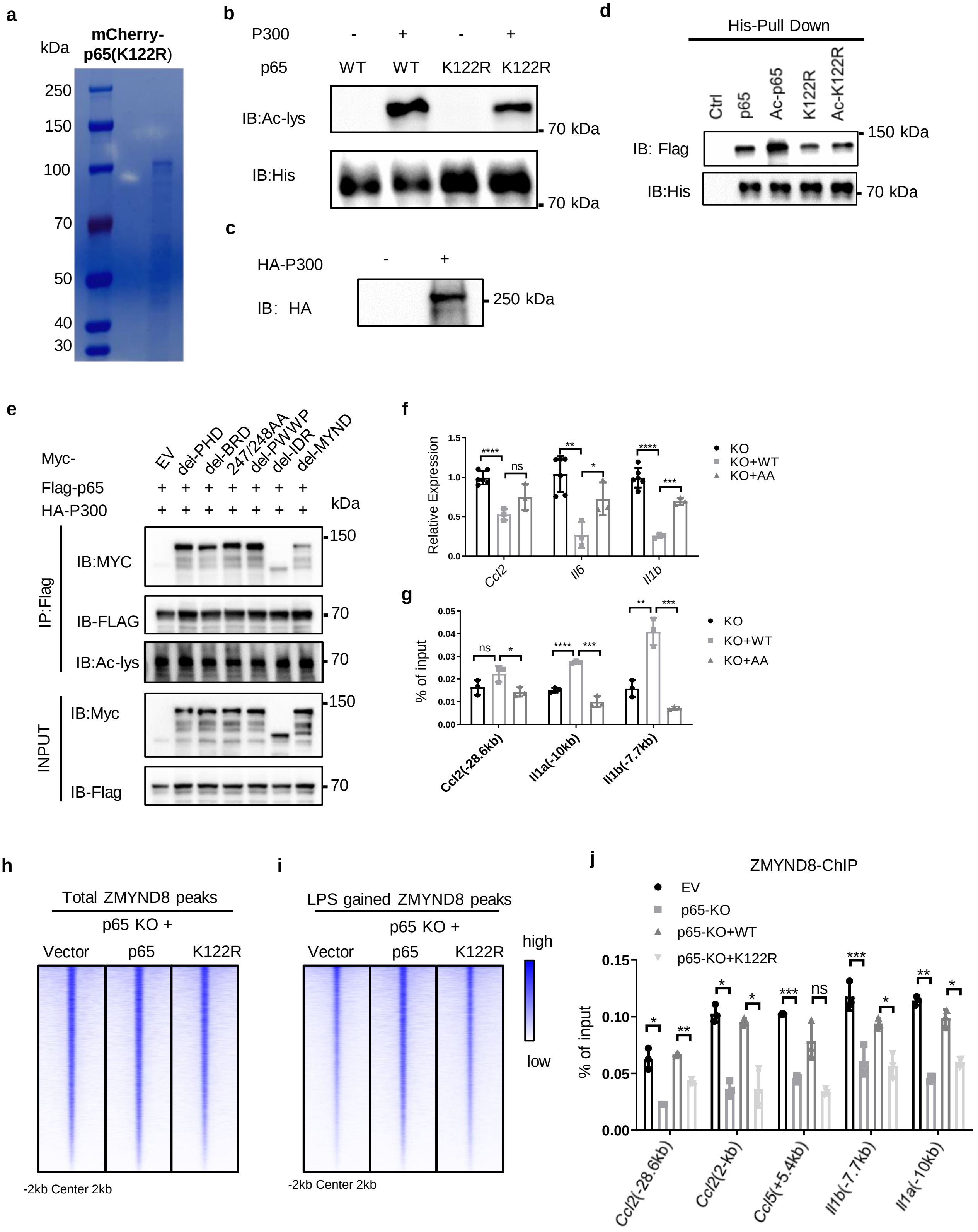

Supplementary Fig. 7 K122 acetylated p65 facilitates the dynamic redistribution of ZMYND8.

(a) p65 K122R mutant expressed and purified from E. coli Rosetta (DE3) cells was evaluated by SDS–PAGE followed by Coomassie blue staining. **(b)** Purified mCherry-p65 and mCherry-p65 K122R mutant were incubated with or without acetyltransferase p300 overexpressed in 293T cells in reaction buffer for 1 hour, and then protein acetylation was analyzed by western blot. **(c)** The expression of acetyltransferase p300. **(d)** His-pull down assay of acetylated p65 *in vitro*. Flag-tagged ZMYND8 immuno-precipitates from transfected HEK293T cells were incubated with nonacetylated or acetylated, His-tagged, WT p65 and K122R mutant. The interaction between ZMYND8 and acetylated p65 was measured by His-Pull down assay. **(e)** IP-immunoblotting of cell lysates prepared from HEK293T cells transfected with indicated vectors for 48 h. The experiments above were repeated three times. **(f)** After six hours of LPS treatment, the relative expression of indicated pro-inflammatory genes was evaluated in different Raw264.7 cells, including *Zmynd8* KO, *Zmynd8* KO + WT *Zmynd8*, and *Zmynd8* KO + (Y247A/N248A) AA mutated cells by qRT-PCR. *$p<0.05$, **$p<0.01$, ***$p < 0.001$, ****$p<0.0001$, ns: not significant, two-tailed Student's *t*-test. Data are presented as mean values +/- SD (n=3 biologically independent samples). **(g)** ChIP-qPCR analysis of ZMYND8 binding intensities at indicated SE regions in different Raw264.7 cells, including *Zmynd8* KO, *Zmynd8* KO + WT *Zmynd8*, and *Zmynd8* KO + AA mutant. *$p<0.05$, **$p<0.01$, ***$p < 0.001$, ****$p<0.0001$, ns: not significant, two-tailed Student's *t*-test. Data are presented as mean values +/- SD (n= 3 biologically independent samples). **(h-i)** Heatmap analyses of the total **(h)** and LPS-gained **(i)** ChIP-seq signals of ZMYND8 in different types of Raw264.7 cells, including the p65 KO, p65 KO + p65 WT, p65 KO + K122R mutant Raw264.7 cells. All ChIP-seq signals are displayed from -2kb to +2kb surrounding the center of each annotated ZMYND8 peak. **(j)** ChIP-qPCR analysis of ZMYND8 binding intensities at SE regions of indicated genes in different Raw264.7 cells, including control (EV), p65 KO, p65 KO + WT p65, and p65 KO + K122R mutant cells. Data are presented as mean values +/- SD (n=3 biologically independent samples). *$p<0.05$, **$p<0.01$, ***$p < 0.001$, ns: not significant, two-tailed Student's *t*-test. Source data are provided as a Source Data file.

Supplementary Fig. 8 ZMYND8 suppresses the latent SEs through LSD1.

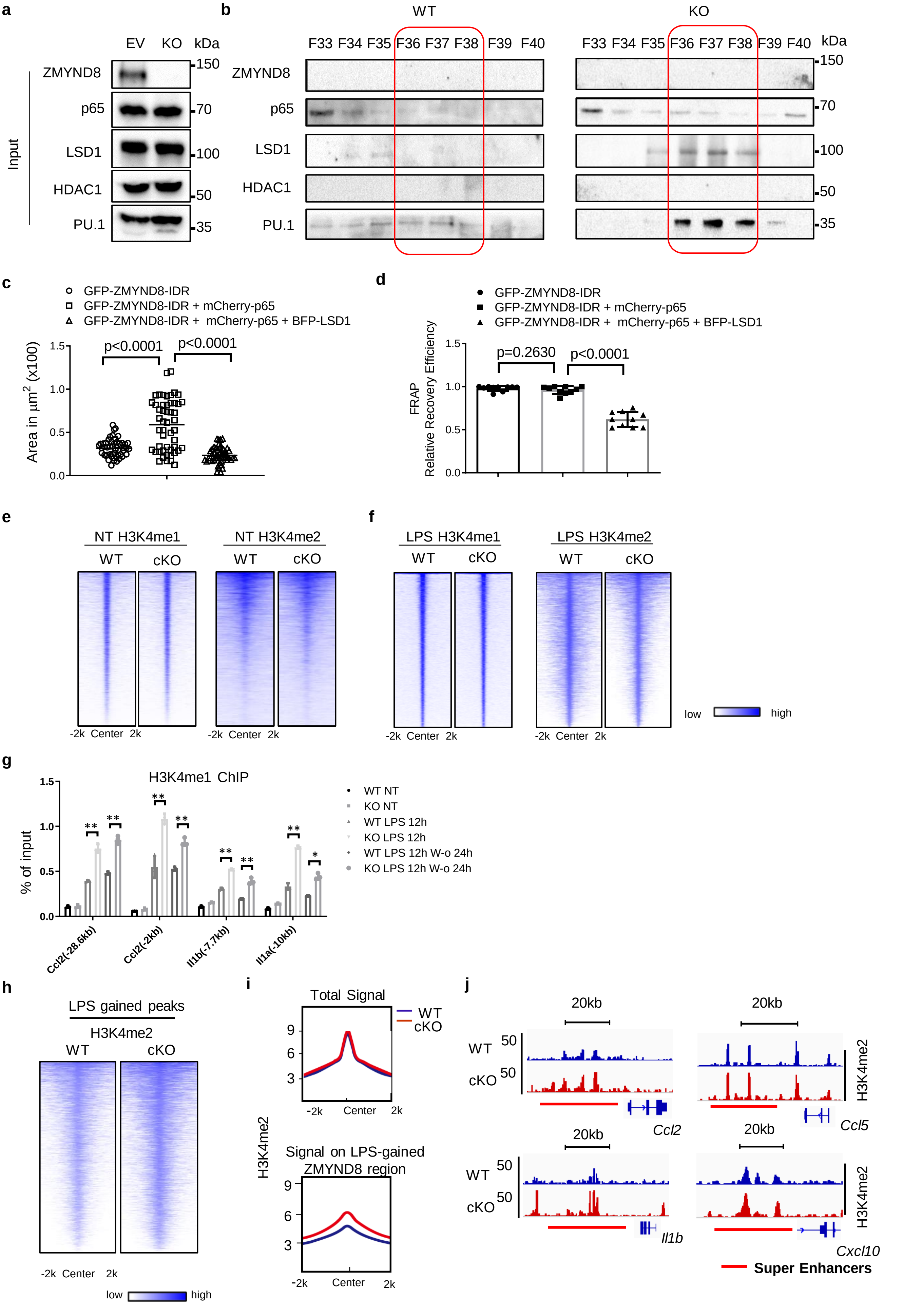

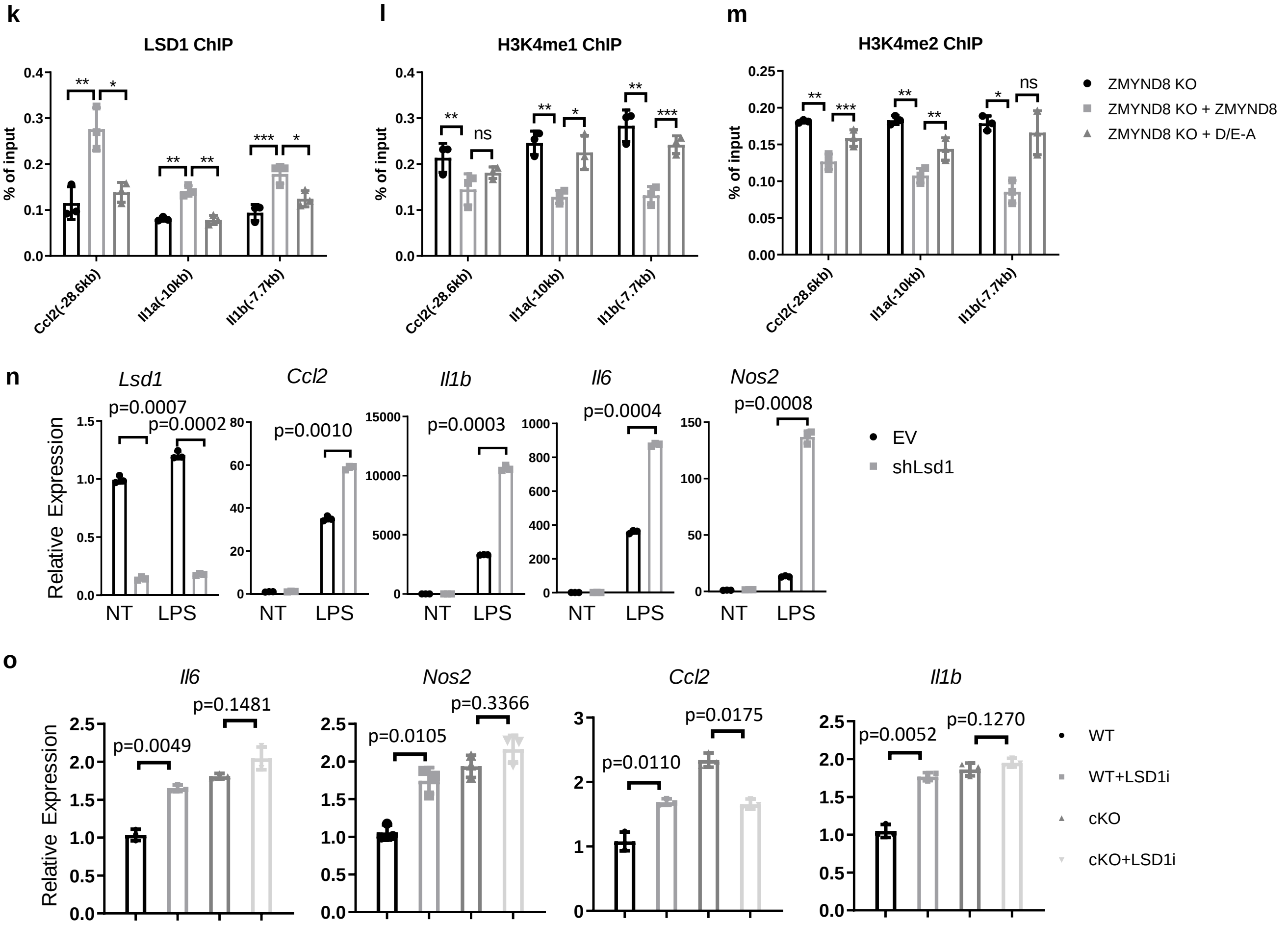
k
LSD1 ChIP
% of input
Ccl2(-28.6kb)
Il1a(-10kb)
Il1b(-7.7kb)
l
H3K4me1 ChIP
% of input
ns
m
H3K4me2 ChIP
% of input
ns
ZMYND8 KO
ZMYND8 KO + ZMYND8
ZMYND8 KO + D/E-A
n
Lsd1
Ccl2
Il1b
Il6
Nos2
Relative Expression
p=0.0007
p=0.0002
p=0.0010
p=0.0003
p=0.0004
p=0.0008
NT
LPS
EV
shLsd1
o
Il6
Nos2
Ccl2
Il1b
Relative Expression
p=0.0049
p=0.1481
p=0.0105
p=0.3366
p=0.0110
p=0.0175
p=0.0052
p=0.1270
WT
WT+LSD1i
cKO
cKO+LSD1i

Supplementary Fig. 8 ZMYND8 suppresses the latent SEs through LSD1.

(a) The input of WT and KO samples for gel filtration chromatography analysis. **(b)** Western blots analyzed the indicated protein level in different fractions from WT and *Zmynd8* KO Raw264.7 cells. Experiments were repeated three times. **(c)** Droplet size formed by GFP-ZMYND8-IDR only, GFP-ZMYND8-IDR with mCherry-p65, GFP-ZMYND8-IDR, mCherry-p65, and BFP-LSD1, respectively (n=50 per sample, examined over three independent experiments). *p* values were determined by two-tailed Student's *t*-test. Data are presented as mean values +/- SD. **(d)** FRAP efficiency of liquid droplets formed by GFP-ZMYND8-IDR, GFP-IDR+mCherry-p65, GFP-IDR+mCherry-p65+BFP-LSD1, respectively (n=10, examined over three independent experiments). *p* values were determined by two-tailed Student's *t*-test. Data are presented as mean values +/- SD. **(e-f**) The H3K4me1 and H3K4me2 levels in untreated (NT) WT and cKO BMDMs **(e)** and LPS-treated BMDMs **(f)** were evaluated by ChIP-Seq. **(g)** ChIP-qPCR analysis of H3K4me1 modification at the SE regions of indicated genes in WT and cKO BMDMs, which were primed with LPS for 12 hours, and then washed away LPS for another 24 hours (n= 3 biologically independent samples). All data are means ± SD. *p* values were determined by two-tailed Student's t test. *$p < 0.05$, **$p < 0.01$. **(h)** Heatmap analysis of the colocalization of H3K4me2 ChIP-Seq signals at LPS-gained ZMYND8 peaks. **(i)** Genome-wide H3K4me2 levels in the WT and *Zmynd8* cKO BMDMs after LPS treatment were evaluated by ChIP-Seq. Histogram plot showing the average distribution of total H3K4me2 (top) and H3K4me2 signals at the LPS-gained ZMYND8 peaks region (bottom). All ChIP-seq signals are displayed from -2kb to +2kb surrounding the center of each annotated H3K4me2 peak. **(j)** Genome browser view of H3K4me2 ChIP-seq peaks at indicated SE regions in WT and cKO BMDMs. **(k-m)** ChIP-qPCR analysis of LSD1 **(k)**, H3K4me1 **(l)** and H3K4me2 **(m)** modification at the SE regions of indicated genes in *Zmynd8* KO rescued with Vector (*Zmynd8* KO + Vector), *Zmynd8* KO with WT *Zmynd8* (*Zmynd8* KO + WT *Zmynd8*) and *Zmynd8* KO with D/E-A mutant (*Zmynd8* KO + D/E-A mutant) Raw264.7 cells (n= 3 biologically independent samples). *$p < 0.05$, **$p < 0.01$, and ***$p < 0.001$, ns: not significant, two-tailed Student's *t*-test. All data are means ± SD. **(n)** RT-qPCR analysis of indicated genes expression in control (EV) and *Lsd1* knockdown (sh-*Lsd1*) Raw264.7 cells treated with LPS or not (NT) (n= 3 biologically independent samples). **(o)** WT and *Zmynd8* cKO BMDMs were pre-treated with vehicle or ORY-100 (LSD1 inhibitor) for 2 hours. Then RT-qPCR analysis of indicated gene expression in LPS treated or untreated (NT) cells (n= 3 biologically independent samples). Two-tailed Student's *t*-test. All data are means ± SD. Source data are provided as a Source Data file.
