# Supplementary material for: ZMYND8 mediated liquid condensates spatiotemporally decommission the latent super-enhancers during macrophage polarization
Source: Nat Commun. 2021 Nov 11;12:6535. doi: 10.1038/s41467-021-26864-x (PMC8586003; doi:10.1038/s41467-021-26864-x)
Supplement: Supplementary file 3 — Description of additional Supplementary File [file 41467_2021_26864_MOESM3_ESM.pdf]

Description of additional Supplementary data files

File name : Supplementary Data 1

Description: The source of plasmids, as well as all the cloning primers and RT-PCR primers, are listed here.
