# Supplementary material for: ZMYND8 mediated liquid condensates spatiotemporally decommission the latent super-enhancers during macrophage polarization
Source: Nat Commun. 2021 Nov 11;12:6535. doi: 10.1038/s41467-021-26864-x (PMC8586003; doi:10.1038/s41467-021-26864-x)
Supplement: Supplementary file 5 — Reporting Summary [file 41467_2021_26864_MOESM5_ESM.pdf]

nature portfolio

Corresponding author(s): Wei Lu

Last updated by author(s): Oct 26, 2021

Reporting Summary

Nature Portfolio wishes to improve the reproducibility of the work that we publish. This form provides structure for consistency and transparency in reporting. For further information on Nature Portfolio policies, see our Editorial Policies and the Editorial Policy Checklist.

Statistics

For all statistical analyses, confirm that the following items are present in the figure legend, table legend, main text, or Methods section.

n/a	Confirmed	
☐	☒	The exact sample size (*n*) for each experimental group/condition, given as a discrete number and unit of measurement
☐	☒	A statement on whether measurements were taken from distinct samples or whether the same sample was measured repeatedly
☐	☒	The statistical test(s) used AND whether they are one- or two-sided<br>*Only common tests should be described solely by name; describe more complex techniques in the Methods section.*
☒	☐	A description of all covariates tested
☒	☐	A description of any assumptions or corrections, such as tests of normality and adjustment for multiple comparisons
☐	☒	A full description of the statistical parameters including central tendency (e.g. means) or other basic estimates (e.g. regression coefficient) AND variation (e.g. standard deviation) or associated estimates of uncertainty (e.g. confidence intervals)
☐	☒	For null hypothesis testing, the test statistic (e.g. *F*, *t*, *r*) with confidence intervals, effect sizes, degrees of freedom and *P* value noted<br>*Give P values as exact values whenever suitable.*
☒	☐	For Bayesian analysis, information on the choice of priors and Markov chain Monte Carlo settings
☒	☐	For hierarchical and complex designs, identification of the appropriate level for tests and full reporting of outcomes
☐	☒	Estimates of effect sizes (e.g. Cohen's *d*, Pearson's *r*), indicating how they were calculated

Our web collection on statistics for biologists contains articles on many of the points above.

Software and code

Policy information about availability of computer code

Data collection	The Flow cytometric data:Beckman Gallios<br>The RNA Sequencing data: Illumina Hiseq 4000<br>The ChIP Sequencing data:Hiseq 2500<br>THe ATAC Sequencing data:NovaSeq 6000<br>The qRT-PCR data :QuantStudio 7 Flex Real Real-Time PCR System<br>The Phase Separation data:LSM880NLO FLIM
Data analysis	Statistical analysis:GraphPad Prism(ver.8)<br>Flow cytometric analysis:Flowjo(Version 10)<br>Heatmap analyses of RNA Sequencing data : RStudio.<br>Confocol data analysis: Image J (2.1.0)<br>Rank Ordering of Super-Enhancers (ROSE) algorithm: https://bitbucket.org/young_computati on/

For manuscripts utilizing custom algorithms or software that are central to the research but not yet described in published literature, software must be made available to editors and reviewers. We strongly encourage code deposition in a community repository (e.g. GitHub). See the Nature Portfolio guidelines for submitting code & software for further information.

Data

Policy information about availability of data

All manuscripts must include a data availability statement. This statement should provide the following information, where applicable:

- Accession codes, unique identifiers, or web links for publicly available datasets
- A description of any restrictions on data availability
- For clinical datasets or third party data, please ensure that the statement adheres to our policy

The RNA-Sequencing data and ChIP-sequencing data have been deposited into the Gene Expression Omnibus(accession code GSE169470).The source data underlying for the main figures and supplementary figures ara provided as a Source Data file,which is included in the submission.

Field-specific reporting

Please select the one below that is the best fit for your research. If you are not sure, read the appropriate sections before making your selection.

☒ Life sciences ☐ Behavioural & social sciences ☐ Ecological, evolutionary & environmental sciences

For a reference copy of the document with all sections, see nature.com/documents/nr-reporting-summary-flat.pdf

Life sciences study design

All studies must disclose on these points even when the disclosure is negative.

Sample size	No sample-size calculation was performed.There are more than in pairs of 10 mice in all animal models.The sample size is sufficient for statistical analysis in the research project. Sample sizes were based on common standards in the field.
Data exclusions	No data were excluded from the analysis.
Replication	All the experiments were three biological replicates. All attempts at replication were successful.
Randomization	All samples were allocated in random.
Blinding	The investigators were blinded to group allocation during data collection and analysis.

Reporting for specific materials, systems and methods

We require information from authors about some types of materials, experimental systems and methods used in many studies. Here, indicate whether each material, system or method listed is relevant to your study. If you are not sure if a list item applies to your research, read the appropriate section before selecting a response.

Materials & experimental systems

n/a	Involved in the study	
☐	☒	Antibodies
☐	☒	Eukaryotic cell lines
☒	☐	Palaeontology and archaeology
☐	☒	Animals and other organisms
☒	☐	Human research participants
☒	☐	Clinical data
☒	☐	Dual use research of concern

Methods

n/a	Involved in the study	
☐	☒	ChIP-seq
☐	☒	Flow cytometry
☒	☐	MRI-based neuroimaging

Antibodies

Antibodies used

Western blot , Confocal and ChIP
ZMYND8 antibody ChIP : Bethyl A302-090A , CHIP: 2ug/sample
ZMYND8 antibody WB : Sigma HPA020949, 1:1000
LSD1 antibody : Abcam ab129195, 2ug/sample, WB 1:1000
H3K4me1 antibody : Cell signaling technology 5326s, 2ug.sample
H3K4me2 antibody : Abcam ab32356, 2ug/sample
p65 antibody : Santa Cruz sc-8008, 2ug/sample, WB 1:1000
MYC tag antibody : Santa Cruz sc-40 HRP, 1:2000
His-Tag antibody : Santa Cruz sc-8036 1:1000
HA-Tag antibody : Santa Cruz sc-7392 1:1000

FLAG tag antibody : Sigma B3111 1:1000
actin antibody : Santa Cruz sc-8432 1:2000
Lamin B1 antibody : Santa Cruz sc-374015 1:1000
Acetylated-Lysine antibody : Cell signaling technology 9441s 1:1000
GFP antibody: Cell signaling technology 2555s 1:1000
HDAC1 antibody : Santa Cruz sc-81598 1:1000
PU.1 antibody : Santa Cruz sc-390405 1:1000
KDM5C antibody : Santa Cruz sc-376255 1:1000
p38 MAPK (D13E1) antibody : Cell signaling technology 8690s 1:1000
Phospho-p38 MAPK (Thr180/Tyr182) (D3F9) : Cell signaling technology 4511s 1:1000
p44/42 MAPK (Erk1/2) (137F5) : Cell signaling technology 4695s 1:1000
Phospho-p44/42 MAPK (Erk1/2) (Thr202/Tyr204) (D13.14.4E) : Cell signaling technology 1:1000
SAPK/JNK antibody : Cell signaling technology 9252s 1:1000
Phospho-SAPK/JNK (Thr183/Tyr185) (G9) : Cell signaling technology 9255s 1:1000
Phospho-NF-κB p65 (Ser536) (93H1) : Cell signaling technology 3033s 1:1000

Secondary antibody
Peroxidase AffiniPure Goat Anti-Rabbit IgG (H+L) : Jackson ImmunoResearch 111-035-003 1:5000
Peroxidase AffiniPure Goat Anti-Mouse IgG (H+L) : Jackson ImmunoResearch 115-035-003 1:5000
Goat anti-Rabbit IgG (H+L) Cross-Adsorbed Secondary Antibody, Alexa Fluor 488 : Invitrogen A-11008 1:400
Goat anti-Mouse IgG (H+L) Cross-Adsorbed Secondary Antibody, Alexa Fluor 594 : Invitrogen A-11005 1:400

Flow cytometry
CD11b-BV421 : Biolegend 101251 (clone:M1/70) 1:300
F4/80-APC : eBioscience 17-4801-80 (clone:BM8) 1:300
Gr-1-PE-CY7 : eBioscience 25-5931-81 (clone:RB6-8C5) 1:300
CD11c-FITC : Invitrogen 11-0114-85 (clone:N418) 1:300
Fc receptor antibody : Biolegend 101302 (clone:93) 1:300

Validation

ZMYND8 Antibody (A302-090A) in IP Detection of human ZMYND8 by western blot of immunoprecipitates. Samples: Whole cell lysate (1 mg for IP, 20% of IP loaded) from HeLa cells. Antibodies: Affinity purified rabbit anti-ZMYND8 antibody A302-090A used for IP at 3 µg/mg lysate. ZMYND8 was also immunoprecipitated by rabbit anti-ZMYND8 antibody A302-089A, which recognizes an upstream epitope. For blotting immunoprecipitated ZMYND8, A302-089A was used at 1 µg/ml. Detection: Chemiluminescence with an exposure time of 10 seconds.

Western blot , Confocal and ChIP
ZMYND8 antibody ChIP : Bethyl A302-090A https://www.thermofisher.cn/cn/zh/antibody/product/PRKCBP1-Antibody-Polyclonal/A302-090A
ZMYND8 antibody WB : Sigma https://www.sigmaaldrich.cn/CN/en/product/sigma/hpa020949?context=product
LSD1 antibody : Abcam ab129195 https://www.abcam.com/kdm1lsd1-antibody-epr6825-nuclear-marker-and-chip-grade-ab129195.html
H3K4me1 antibody : Cell signaling technology 5326s https://www.cellsignal.com/products/primary-antibodies/mono-methyl-histone-h3-lys4-d1a9-xp-rabbit-mab/5326
H3K4me2 antibody : Abcam ab32356 https://www.abcam.com/Histone-H3-di-methyl-K4-antibody-Y47-ChIP-Grade-ab32356.html
p65 antibody : Santa Cruz sc-8008 https://www.scbt.com/zh/p/nfkappab-p65-antibody-f-6
MYC tag antibody : Santa Cruz sc-40 HRP https://www.scbt.com/p/c-myc-antibody-9e10?requestFrom=search
His-Tag antibody : Santa Cruz sc-8036 https://www.scbt.com/zh/p/his-probe-antibody-h-3?requestFrom=search
HA-Tag antibody : Santa Cruz sc-7392 https://www.scbt.com/zh/p/ha-probe-antibody-f-7?requestFrom=search
FLAG tag antibody : Sigma B3111 https://www.sigmaaldrich.cn/CN/en/product/sigma/b3111?context=product
actin antibody : Santa Cruz sc-8432 https://www.scbt.com/p/actin-antibody-c-2?requestFrom=search
Lamin B1 antibody : Santa Cruz sc-374015 https://www.scbt.com/zh/p/lamin-b1-antibody-b-10?requestFrom=search
Acetylated-Lysine antibody : Cell signaling technology 9441s https://www.cellsignal.com/products/primary-antibodies/acetylated-lysine-antibody/9441
GFP antibody: Cell signaling technology 2555s https://www.cellsignal.com/products/primary-antibodies/gfp-antibody/2555
HDAC1 antibody : Santa Cruz sc-81598 https://www.scbt.com/zh/p/hdac1-antibody-10e2?requestFrom=search
PU.1 antibody : Santa Cruz sc-390405 https://www.scbt.com/p/pu-1-antibody-c-3?requestFrom=search
KDM5C antibody : Santa Cruz sc-376255 https://www.scbt.com/zh/p/smcx-antibody-g-10?requestFrom=search
p38 MAPK (D13E1) antibody : Cell signaling technology 8690s https://www.cellsignal.com/products/primary-antibodies/p38-mapk-d13e1-xp-rabbit-mab/8690?site-search-type=Products&N=4294956287&Ntt=p38&fromPage=plp
Phospho-p38 MAPK (Thr180/Tyr182) (D3F9) : Cell signaling technology 4511s https://www.cellsignal.com/products/primary-antibodies/phospho-p38-mapk-thr180-tyr182-d3f9-xp-rabbit-mab/4511?site-search-type=Products&N=4294956287&Ntt=phospho-p38&fromPage=plp
p44/42 MAPK (Erk1/2) (137F5) : Cell signaling technology 4695s https://www.cellsignal.com/products/primary-antibodies/p44-42-mapk-erk1-2-137f5-rabbit-mab/4695?site-search-type=Products&N=4294956287&Ntt=erk&fromPage=plp
Phospho-p44/42 MAPK (Erk1/2) (Thr202/Tyr204) (D13.14.4E) : Cell signaling technology 4370 https://www.cellsignal.com/products/primary-antibodies/phospho-p44-42-mapk-erk1-2-thr202-tyr204-d13-14-4e-xp-rabbit-mab/4370?site-search-type=Products&N=4294956287&Ntt=erk&fromPage=plp
SAPK/JNK antibody : Cell signaling technology 9252 https://www.cellsignal.com/products/primary-antibodies/sapk-jnk-antibody/9252
Phospho-SAPK/JNK (Thr183/Tyr185) (G9) : Cell signaling technology 9255 https://www.cellsignal.com/products/primary-antibodies/phospho-sapk-jnk-thr183-tyr185-g9-mouse-mab/9255?site-search-type=Products&N=4294956287&Ntt=jnk&fromPage=plp

Phospho-NF-κB p65 (Ser536) (93H1) : Cell signaling technology 3033s https://www.cellsignal.com/products/primary-antibodies/phospho-nf-kb-p65-ser536-93h1-rabbit-mab/3033

Flow cytometry
CD11b-BV421 : Biolegend 101251 (clone:M1/70) https://www.biolegend.com/en-us/products/brilliant-violet-421-anti-mouse-human-cd11b-antibody-7163?GroupID=BLG10427
F4/80-APC : eBioscience 17-4801-80 (clone:BM8) https://www.thermofisher.cn/cn/zh/antibody/product/F4-80-Antibody-clone-BM8-Monoclonal/17-4801-82?adobe_mc=MCMID%7C26088330770613422071955603836484247741%7CMCAID%3D301A4E087FE22D69-4000030F90CBD596%7CMCORGID%3D5B135A0C5370E6B40A490D44%40AdobeOrg%7CTS=1614293705
Gr-1-PE-CY7 : eBioscience 25-5931-81 (clone:RB6-8C5) https://www.thermofisher.cn/cn/zh/antibody/product/Ly-6G-Ly-6C-Antibody-clone-RB6-8C5-Monoclonal/25-5931-82?adobe_mc=MCMID%7C26088330770613422071955603836484247741%7CMCAID%3D301A4E087FE22D69-4000030F90CBD596%7CMCORGID%3D5B135A0C5370E6B40A490D44%40AdobeOrg%7CTS=1614293705
CD11c-FITC : Invitrogen https://www.thermofisher.cn/cn/zh/antibody/product/CD11c-Antibody-clone-N418-Monoclonal/11-0114-85

Eukaryotic cell lines

Policy information about cell lines

Cell line source(s)	Raw264.7 cells,HEK293T cells and HeLa cells were purcased from Cell bank of Chinese Academic of Sciences.
Authentication	All cell lines were authenticated by observing the morphology.
Mycoplasma contamination	There is no mycoplasma contamination in all cell lines.
Commonly misidentified lines (See ICLAC register)	No commonly misidentified cell lines were used.

Animals and other organisms

Policy information about studies involving animals; ARRIVE guidelines recommended for reporting animal research

Laboratory animals	The Zmynd8flox/flox mouse model was established by gene targeting via homologous recombination in C57BL/6 embryonic stem cells (Cygene, Guangzhou, China). LoxP sequences were introduced to flank exons 6 and 7 of the Zmynd8 gene. Zmynd8flox/flox mice were crossed with specific Cre transgenic mice to achieve lineage-specific gene deletion. Lyz2-Cre (also known as LysM-Cre) mice on a C57BL/6 background were purchased from the Jackson Laboratory.<br>Mice were housed under specific-pathogen-free conditions with standard food and water ad libitum in a 12h light / 12h dark cycle. Humidity and ambient temperature were maintained between 45-65% and 20-24°C, respectively.<br>6-week-old female C57BL/6 mice were used.
Wild animals	The study did not involve wild animals.
Field-collected samples	The study did not involve sample collected from the filed.
Ethics oversight	Mouse experimental protocols were approved by the Institutional biomedical research ethics committee of Shanghai Institute of Nutrition and Health Sciences, Chinese Academy of Sciences.

Note that full information on the approval of the study protocol must also be provided in the manuscript.

ChIP-seq

Data deposition

☒ Confirm that both raw and final processed data have been deposited in a public database such as GEO.

☒ Confirm that you have deposited or provided access to graph files (e.g. BED files) for the called peaks.

Data access links<br>*May remain private before publication.*	To review GEO accession GSE169470:<br>Go to https://www.ncbi.nlm.nih.gov/geo/query/acc.cgi?acc=GSE169470
Files in database submission	GSE169470 including the below files:<br>Input<br>M0_ZMYND8<br>M1_ZMYND8<br>Input-0% 1,6-hex_BMRC200002491-1A_1.clean.fq<br>Input-0% 1,6-hex_BMRC200002491-1A_2.clean.fq<br>Input-6% 1-6-Hex_BMRC200002492-1A_1.clean.fq<br>Input-6% 1-6-Hex_BMRC200002492-1A_2.clean.fq<br>0% 1,6-hex_BMRC200002500-1A_1.clean.fq<br>0% 1,6-hex_BMRC200002500-1A_2.clean.fq<br>6% 1-6-Hex_BMRC200002493-1A_1.clean.fq

6% 1-6-Hex_BMRC200002493-1A_2.clean.fq
Input WT(H3K4me1)_FMRC202440229-1a_1.clean.fq
Input WT(H3K4me1)_FMRC202440229-1a_2.clean.fq
Input-Z8 KO(H3K4me1)_FMRC202440230-1b_1.clean.fq
Input-Z8 KO(H3K4me1)_FMRC202440230-1b_2.clean.fq
WT-H3K4me1_FMRC202440233-1a_1.clean.fq
WT-H3K4me1_FMRC202440233-1a_2.clean.fq
Z8 KO-H3K4me1_FMRC202440234-1a_1.clean.fq
Z8 KO-H3K4me1_FMRC202440234-1a_2.clean.fq
Input-WT-Lsd1_BMRC200005355-1A_1.clean.fq
Input-WT-Lsd1_BMRC200005355-1A_2.clean.fq
Input-Z8 KO-Lsd1_BMRC200005354-1A_1.clean.fq
Input-Z8 KO-Lsd1_BMRC200005354-1A_2.clean.fq
WT-Lsd1_BMRC200005353-1A_1.clean.fq
WT-Lsd1_BMRC200005353-1A_2.clean.fq
Z8 KO-Lsd1_BMRC200005356-1A_1.clean.fq
Z8 KO-Lsd1_BMRC200005356-1A_2.clean.fq
Input p65 KO LPS_FMRC202450919-1a_1.clean.fq
Input p65 KO LPS_FMRC202450919-1a_2.clean.fq
Input p65 KO +WT LPS_FMRC202450920-1a_1.clean.fq
Input p65 KO +WT LPS_FMRC202450920-1a_2.clean.fq
Input p65 KO+K122R LPS_FMRC202450921-1a_1.clean.fq
Input p65 KO+K122R LPS_FMRC202450921-1a_2.clean.fq
p65 KO_FMRC202450922-1a_1.clean.fq
p65 KO_FMRC202450922-1a_2.clean.fq
p65 KO+WT_FMRC202450923-1a_1.clean.fq
p65 KO+WT_FMRC202450923-1a_2.clean.fq
p65 KO+K122R_FMRC202450924-1a_1.clean.fq
p65 KO+K122R_FMRC202450924-1a_2.clean.fq
Input-Z8 KO-NT_BMRC200002494-1A_1.clean.fq
Input-Z8 KO-NT_BMRC200002494-1A_2.clean.fq
Input-Z8 KO+Z8-NT_BMRC200002495-1A_1.clean.fq
Input-Z8 KO+Z8-NT_BMRC200002495-1A_2.clean.fq
Input-Z8 KO+DE-A-NT_BMRC200002496-1A_1.clean.fq
Input-Z8 KO+DE-A-NT_BMRC200002496-1A_2.clean.fq
Input-Z8 KO-LPS_BMRC200002497-1A_1.clean.fq
Input-Z8 KO-LPS_BMRC200002497-1A_2.clean.fq
Input-Z8 KO+Z8-LPS_BMRC200002498-1A_1.clean.fq
Input-Z8 KO+Z8-LPS_BMRC200002498-1A_2.clean.fq
Input-Z8 KO+DE-A-LPS_BMRC200002499-1A_1.clean.fq
Input-Z8 KO+DE-A-LPS_BMRC200002499-1A_2.clean.fq
Z8 KO-NT_BMRC200002501-1A_1.clean.fq
Z8 KO-NT_BMRC200002501-1A_2.clean.fq
Z8 KO+Z8-NT_BMRC200002502-1A_1.clean.fq
Z8 KO+Z8-NT_BMRC200002502-1A_2.clean.fq
Z8 KO+DE-A-NT_BMRC200002503-1A_1.clean.fq
Z8 KO+DE-A-NT_BMRC200002503-1A_2.clean.fq
Z8 KO-LPS_BMRC200002504-1A_1.clean.fq
Z8 KO-LPS_BMRC200002504-1A_2.clean.fq
Z8 KO+Z8-LPS_BMRC200002505-1A_1.clean.fq
Z8 KO+Z8-LPS_BMRC200002505-1A_2.clean.fq
Z8 KO+DE-A-LPS_BMRC200002506-1A_1.clean.fq
Z8 KO+DE-A-LPS_BMRC200002506-1A_2.clean.fq
H3K4me1_LPS_BMRC210000386-1A_1.clean.fq
H3K4me1_LPS_BMRC210000386-1A_2.clean.fq
H3K4me1_NT_BMRC210000385-1A_1.clean.fq
H3K4me1_NT_BMRC210000385-1A_2.clean.fq
H3K27ac_LPS_BMRC210000388-1A_1.clean.fq
H3K27ac_LPS_BMRC210000388-1A_2.clean.fq
H3K27ac_NT_BMRC210000387-1A_1.clean.fq
H3K27ac_NT_BMRC210000387-1A_2.clean.fq
p65_LPS_BMRC210000384-1A_1.clean.fq
p65_LPS_BMRC210000384-1A_2.clean.fq
p65_NT_BMRC210000383-1A_1.clean.fq
p65_NT_BMRC210000383-1A_2.clean.fq
H3K4me2_KO_BMRC210000391-1A_1.clean.fq
H3K4me2_KO_BMRC210000391-1A_2.clean.fq
H3K4me2_WT_BMRC210000390-1A_1.clean.fq

	H3K4me2_WT_BMRC210000390-1A_2.clean.fq<br>WT_H3K4me1_NT_BMRC210002333-1A_1.clean.fq<br>WT_H3K4me1_NT_BMRC210002333-1A_2.clean.fq<br>WT_H3K4me2_NT_BMRC210002331-1A_1.clean.fq<br>WT_H3K4me2_NT_BMRC210002331-1A_2.clean.fq<br>KO_H3K4me1_NT_BMRC210002334-1A_1.clean.fq<br>KO_H3K4me1_NT_BMRC210002334-1A_2.clean.fq<br>KO_H3K4me2_NT_BMRC210002332-1A_1.clean.fq<br>KO_H3K4me2_NT_BMRC210002332-1A_2.clean.fq<br>Input_KO_BMRC210000389-1A_1.clean.fq<br>Input_KO_BMRC210000389-1A_2.clean.fq<br>Input_WT_BMRC210000378-1A_1.clean.fq<br>Input_WT_BMRC210000378-1A_2.clean.fq<br>Input_LPS_BMRC210000377-1A_1.clean.fq<br>Input_LPS_BMRC210000377-1A_2.clean.fq<br>Input_NT_BMRC210000376-1A_1.clean.fq<br>Input_NT_BMRC210000376-1A_2.clean.fq<br>Input_LPS_BMRC210000377-1A_1.clean.fq<br>Input_LPS_BMRC210000377-1A_2.clean.fq<br>Input_KO_NT_BMRC210002330-1A_1.clean.fq<br>Input_KO_NT_BMRC210002330-1A_2.clean.fq<br>Input_WT_NT_BMRC210002329-1A_1.clean.fq<br>Input_WT_NT_BMRC210002329-1A_2.clean.fq
Genome browser session (e.g. UCSC)	No longer applicable

Methodology

Replicates	ChIP-seq for each group of the cells,which were pooled from three mice,was performed once.
Sequencing depth	The amount of data for each sample is 6G,Reads were aligned to the mouse genome (mm10), and after removal of duplicate and non-uniquely mapped reads, ~8 million alignments were obtained.They are paired-end.
Antibodies	ZMYND8 antibody ChIP-seq Bethyl A302-090A , LSD1 antibody ChIP-seq Abcam ab129195,H3K4me1 antibody ChIP-seq Cell signaling technology 5326s, H3K4me2 antibody ChIP-seq Abcam ab32356, p65 antibody ChIP-seq Santa Cruz sc-8008.
Peak calling parameters	MACS peak finding was performed to identify the most significant peaks. Using our default cutoff of p-value 1e-7 (control file is input file)
Data quality	The ZMYND8（A302-090A，Bethyl）antibody for ChIP-seq was validated by Active Motif. The LSD1(ab129195,Abcam) and H3K4me1(5326s,Cell signaling technology) antibody for ChIP-seq were validated by Novogene.The correct size of chromatin and libraries was checked in a gel.Only raw reads paasing the QC were used for alignment.
Software	We used Galaxy on line analyse website to analyse ChIP-seq data.Visualization was performed in IGV.

Flow Cytometry

Plots

Confirm that:

- [x] The axis labels state the marker and fluorochrome used (e.g. CD4-FITC).
- [x] The axis scales are clearly visible. Include numbers along axes only for bottom left plot of group (a 'group' is an analysis of identical markers).
- [x] All plots are contour plots with outliers or pseudocolor plots.
- [x] A numerical value for number of cells or percentage (with statistics) is provided.

Methodology

Sample preparation	The tissues were ground into a single cell suspension, then stained with flow antibody.<br>For surface staining,cells were stained in PBS containing 2% FBS for 30 minutes at 4 degree,followed by washing by PBS for 2 times prior to Flow Cytometry analysis.
Instrument	Beckman Gallios flow cytometer
Software	Flowjo
Cell population abundance	There is no abundance of the relevant cell populations.

Gating strategy

For all experiments,cells were first gated by FSC/SSC to exclude debris,followed by gating FSC-A and FSC-H to eliminate nonsinglets.Then,target cell population for further analysis were gated by cell surface marker.

☒ Tick this box to confirm that a figure exemplifying the gating strategy is provided in the Supplementary Information.
